# Cardiovascular Diseases Diagnosis by Impedance Cardiography

**DOI:** 10.2478/joeb-2022-0013

**Published:** 2022-12-25

**Authors:** Sofiene Mansouri, Yousef Alharbi, Anwar Alshrouf, Abdulrahman Alqahtani

**Affiliations:** 1 Department of Biomedical Technology, College of Applied Medical Sciences, Prince Sattam bin Abdulaziz University, Al-Kharj, Saudi Arabia; 2 Laboratory of Biophysics and Medical Technologies, Higher Institute of Medical Technologies of Tunis, University of Tunis El Manar, Tunis Tunisia; 3 Department of Medical Equipment Technology, College of Applied Medical Science, Majmaah University, Majmaah City, Saudi Arabia

**Keywords:** CDVs, ICG, Diagnosis

## Abstract

Cardiovascular disease (CVD) represents the leading cause of mortality worldwide. In order to diagnose CVDs, there are a range of detection methods, among them, the impedance cardiography technique (ICG). It is a non-invasive and low-cost method. In this paper, we highlight recent advances and developments of the CDVs diagnosis mainly by the ICG method. We considered papers published during the last five years (from 2017 until 2022). Based on this study, we expressed the need for an ICG database for the different CDVs.

## Introduction

Cardiovascular disease (CVD), despite the significant advances in the diagnosis and treatments, still represents the leading cause of mortality worldwide [1]. An estimated 17.9 million people died from CVDs in 2019, representing 32% of all global deaths. Of these deaths, 85% were due to heart attack and stroke. It is important to detect CVD as early as possible so that management with counselling and medicines can begin [2].

In order to diagnose CVDs, there is a range of detection methods and medical devices at the disposal of doctors: X-ray machine, computed tomography, doppler-based echocardiography, echocardiography, electrocardiography, magnetic resonance imaging, Holter monitoring, stress test, cardiac catheterization, blood tests, coronary angiography, and radionuclides tests [3, 4, 5].

The bioimpedance technique is very useful and advantageous in the medical field because it is non-invasive, flexible, simple, reliable, safe, painless, at low cost, manipulable by a nurse or technician, and fast, thus saving time for better care and effective intervention on the patient. Electrical bioimpedance studies the impedance in the human body. It allows obtaining continuous and real-time hemodynamic data measurements as well as the diagnosis of several cardiovascular diseases, in particular by using the impedance cardiography (ICG) technique. It also provides a better distribution where the noises are minimal, and the electrode-skin surface impedance is low [6].

The main purpose of this paper is to highlight the main recent advances and developments of the CDVs diagnosis by the ICG method. We considered papers published during the last five years (from 2017 until 2022). The second objective is to draw scientific community attention to the need and necessity of having a database of ICG signals.

In the next sections, we presented the bioimpedance method, the cardiovascular parameters, the CDVs diagnosis, limitations, and possible improvements.

## Electrical bioimpedance

Fundamentals, recent advances, and future challenges in bioimpedance devices for healthcare applications have been well introduced by D. Naranjo-Hernández et al. [7].

**Fig. 1:**
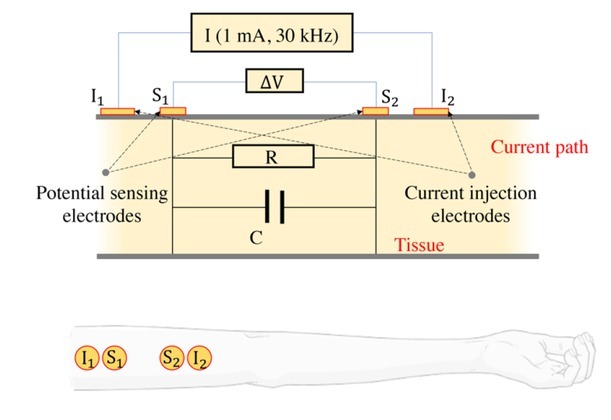
Electrical bioimpedance electrodes configuration.

In the body, resistance varies according to the nature of the tissue; lean tissues containing large amounts of water represent a path of low electrical resistance. On the other hand, fat and bones are poor conductors (high electrical resistance) with small amounts of fluids. Electrical bioimpedance is also a non-invasive method of measuring the change in a blood volume at the level of a body segment. This technique is low cost, safe, reliable, painless, and real time. Bioimpedance consists in exploring the thorax [8], this is thoracic bioimpedance or impedance cardiography (ICG), Fig. 1.a, or limbs [9], Fig. 1.b, which is then the peripheral bioimpedance.

In general, a constant, “high frequency” and low intensity current is applied, through two electrodes placed in the front of and above the leading edge of the heart.

The measurement voltage is acquired through two other electrodes placed on the chest of the patient between the injection electrodes (in the left side of the patient's chest). The electrode configuration for recording the bioimpedance signal is shown in Fig. 1. During the recording process, the patient must lie supine, relaxed and in expiratory apnea for a period of 10 s [10].

**Fig. 2:**
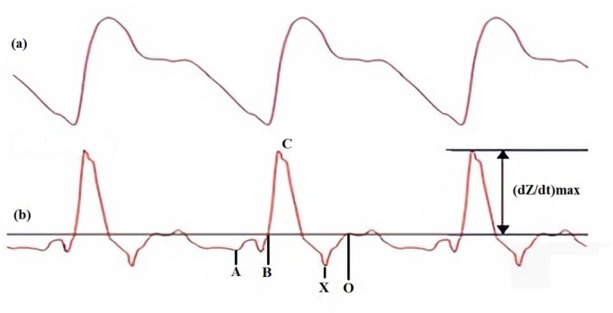
(a) The top plot represents the bioimpedance signal (ΔZ) (change in impedance over time). (b) The bottom plot represents the ICG signal (the first derivative of the bioimpedance signal)

According to Ohm's law, the variation in impedance (ΔZ) of the body section explored is the ratio between the variation in the voltage acquired and the current injected. The plot showing the change in impedance over time is called the bioimpedance signal (ΔZ) (Fig. 2.a). This signal is characterized by an ascending phase corresponding to systolic ejection and a descending phase corresponding to the ventricular diastole, followed by a dicrotic wave corresponding to the closure of the aortic valve, then a general diastole.

The first derivative of the bioimpedance signal (ΔZ) is called the ICG signal denoted $\frac{dz}{dt}$(Fig. 2.b).

The ICG signal is characterized by five important points: A, B, C, X and O, corresponding to distinct physiological events in the cardiac cycle [11]_._

Point A coincides with the start of systole. It is associated with atrial contraction; its amplitude is correlated with the ejection fraction of the left atrium.

Point B appears simultaneously with the opening of the aortic valve. Identifying this point is very important for the calculation of systolic ejection duration and cardiac output. Point C (also called point E) corresponds to the peak of the ICG signal. It is associated with ventricular contraction (corresponding to the ejection of blood from the ventricles). The amplitude of C is mainly used to determine stroke volume and cardiac output. ${\left(\frac{dZ}{dt}\right)}_{\max}$is the maximum amplitude of the signal, measured from the baseline to point C.

Point X is the lowest point in the negative part of the ICG signal after point C, it is associated with the closure of the aortic valve. This point indicates the end of the systole.

Point O is associated with the change in volume during the diastolic phase and with the maximum opening of the mitral valve [11]_._

Bioimpedance may be useful for diagnosis and treatment in different clinical situations such as CVDs, hypertension, dyspnea, mechanical ventilation, dialysis and early detection of skin or breast cancer [12]. In addition, bioimpedance can be used for the assessment of hemodynamic parameters in patients under anesthesia, patients under mechanical ventilation as well as during different surgical operations at different patient position [13].

The ICG signal can be measured by systems like Cardio Dynamics BioZ ICG Hemodynamic Monitor [14], NiCaS, NI Medical [15], Niccomo [16], FhysioFlow Manatec Biomedical [17], HIC-2000 Bio-electric Impedance Cardiograph from Bio-Impedance Technology [18], and Cardiotronic Osypka Medical [19]. There also devices that can calculate the stroke volume (SV) and more than 30 hemodynamic parameters, CardioScreen 2000 [20], and CardioScreen 1000 [21].

The ICG signal is usually used to calculate hemodynamic parameters. Measurement by ICG has been strongly correlated with echocardiography [22, 23], thermodilution [24, 25], and the Fick method [26].

## Cardiovascular parameters

ICG was introduced as a noninvasive method. Its waveform has been used for the assessment of certain hemodynamic parameters describing the mechanical function of the heart. These parameters include cardiac output (CO), SV, and systolic time intervals, e.g., left ventricular ejection time (LVET noted also ET), preejection period (PEP), and systolic time ratio (STR) [27].

ICG signals also help in determination of left ventricular end systolic volume (LVESV), left ventricular end diastolic volume (LVEDV), left ventricular ejection fraction (LVEF), iso volumetric contraction time (IVCT), iso volumetric relaxation time (IVRT), total systolic time (TST), total diastolic time (TDT), and myocardial performance index (MPI), with minimal errors [3].

We determine also the thoracic base impedance (Z_0_) during the diastole considered constant for a patient at about 25 Ω, for a man from 20 to 33 Ω and a woman from 27 to 48 Ω [28].

The bioimpedance method can also successfully detect respiration rate and heart rate [13, 29].

We can also deduce arterial compliance, which is an important cardiovascular risk factor [30], and can be used as a predictor of future heart disease and also in patients with diabetes [31]. Compliance is a measure of the elastic properties of arterial vessels [32]. The ability of a vessel to distend and increase volume with increasing transmural pressure is quantified as vessel compliance (C), which is the change in volume (ΔV) divided by the change in pressure (ΔP) [32]. Precisely, arterial compliance is described as the ratio between the volume variation and the pressure variation (ΔV/ΔP). Therefore, a high blood pressure (hypertension) can be due to a decrease in arterial compliance [33]. For a given quantity of blood pressure, vessels with a high compliance can be filled with large volumes whereas vessels with low compliance can only be filled with small volumes [34].

Another important prognostic tool in clinical practice is the phase angle (PhA) of bioimpedance. The PhA is used as an indicator of cellular health, particularly reflecting cell membrane integrity and cell function [35]. Authors have investigated the association between phase angle and CVDs and the values of the phase angle were lower in individuals with CVD than in control subjects [36].

The bioimpedance phase angle has been considered as a predictor for morbidity and mortality in different clinical situations. The reference values for mean phase angle values in healthy individuals are estimated from this meta-analysis. In both sexes, phase-angle values have a similar pattern that starts from infants, increase progressively up to the teenage phase, stabilize during adult ages, and then decrease progressively in older subjects and the elderly. Males have higher estimates than females for all age groups except for infants (0-2) and subjects older than 80 years old [37].

In another systematic review and meta-analysis, the positive association of physical activity with phase angle reinforces the importance of routinely including exercise in health care [38, 39].

## Diagnosis of CDVs

In order to diagnose CVDs, there are a range of detection methods and medical devices at the disposal of doctors when they suspect a patient may have heart disease [5]. X-ray machine, computed tomography, doppler based echocardiography, echocardiography, electrocardiography, magnetic resonance imaging, Holter monitoring, stress test, cardiac catheterization, blood tests, coronary angiography, and radionuclides tests [3].

ICG can be useful and reliable for diagnosing different CVDs and for therapeutic decisions-making. In this section, we presented the detection and diagnosis by using the ICG method of the valvular heart diseases, cardiac arrhythmias, hypertensive heart diseases, vascular diseases, heart failure and different other diseases such as Cushing’s disease.

## Valvular heart disease

Valvular heart disease (VHD) [40] is characterized by the affection of one or more heart valves: mitral [41, 42, 43], aortic [44], tricuspid or pulmonary valves and it is usually diagnosed using Doppler echocardiography.

Chabchoub et al. proposed a new computer aided diagnosis system to detect VHD using the ICG signals. Six types of ICG heartbeats are analyzed and classified: normal heartbeats (N), mitral insufficiency heartbeats (MI), aortic insufficiency heartbeats (AI), mitral stenosis heartbeats (MS), aortic stenosis heartbeats (AS), and pulmonary stenosis heartbeats (PS). The proposed methodology was validated on 120 ICG recordings. Firstly, the ICG signal is denoised using the Daubechies wavelet family with order eight (db8). Then, these signals are segmented into several heartbeats and, later, subjected to the linear prediction LP and discrete wavelet transform DWT approaches to extract 16 temporal and time–frequency features. For the classification step, the support vector machine SVM and k nearest neighbors KNN classifiers are used. Obtained results showed that the combination between the 16 temporal and time– frequency features and SVM classifier achieved the highest classification performance in classifying the N, MI, MS, AI, AS and PS heartbeats with 98.94% of overall accuracy [45].

## Hypertensive heart disease

Hypertension (high blood pressure) is considered as the most common risk-factor for premature CVD. The early detection and the good management of hypertension can reduce the risk of further complications. The ICG technique is widely used in the diagnosis and the management of hypertension [46, 47].

Barochiner et al. [4] investigated the ability of CI, SVRI, blood pressure (BP), and HR parameters derived from the ICG method, to distinguish hypertensive patients with exaggerated orthostatic blood pressure variation (EOV). They noticed a very important raise in SVRI and HR in patients with orthostatic hypotension compared to the patients with normal orthostatic BP variation and orthostatic hypertension.

ICG has shown that ventricular, vascular, and hemodynamic abnormalities are prevalent in adults over 40 years of age with at least 2 cardiovascular (CV) risk factors. This suggests that all stage 1 hypertension patients with multiple CV risk factors, over 40 years of age, have early-stage CV disease and should be treated. For many of these patients, ICG testing has shown low cardiac index with high systemic vascular resistance or vice versa. If only BP is measured, these significant extremes in hemodynamics would be unknown. With ICG data, drug selection and titration can be customized based on the abnormal underlying mechanisms to rapidly control BP while reducing adverse side effects and preventing progression of CV disease [48, 49].

In [50], hypertensive patients with heart failure (HF) had significantly lower values of blood flow parameters, contractility, and left work indices compared with hypertensive patients without HF. These differences reflected the incorrect hemodynamic pattern (mostly hypodynamic) of these patients. ICG seems to be an adequate method to reflect these differences.

## Heart arrhythmia

Ventricular arrhythmias (VAs) are independently related to mortality risk in patients with heart failure (HF). The wide availability of implantable cardioverter defibrillators and cardiac resynchronization therapy devices now offers an opportunity to clinically correlate the two disease processes. Our study found a significant positive relationship between changes in intrathoracic impedance and episodes of VAs in patients with HF [51, 52, 53].

Identifying the optimal atrioventricular (AV) or inter-ventricular (VV) delay is beneficial for patients using cardiac resynchronization therapy (CRT) devices. ICG can calculate stroke volume by measuring changes in transthoracic electric impedance. ICG might be a promising tool for the rapid optimization of cardiac resynchronization therapy devices [54].

## Vascular diseases

The cardiovascular system consists of the heart, a network of vessels and blood. Disorders in blood vessels can cause a range of health problems which can be severe or fatal. The blood pooling method has been used to measure the change in blood volume of the thigh, abdomen, limbs, etc. with the pressure change. This method occludes the vessel to prevent blood flowing out of the segment being investigated.

Authors have shown that vascular diseases can be detected by analyzing the difference in bioimpedance measurements calculated before and after blood pooling. The simulation results clarify the ability of BIA in estimating vascular abnormalities, depending on the changes caused by diseases in vessel compliance, which are reflected in bioimpedance measurements using the blood pooling method [55].

Hammoud et al. [56] presented an approach for determining vascular tone in human extremities based on multi-channel bioimpedance measurements. Vascular tone plays a vital role in regulating blood pressure and coronary circulation, and it determines the peripheral vascular resistance. This study is a key step towards understanding the way vascular tone changes in the extremities and how the nervous system regulates these changes.

Anyfanti et al. [57] studied the association of non-invasive hemodynamics with arterial stiffness in rheumatoid arthritis and conclude that among patients with rheumatoid arthritis, arterial stiffness appears as the composite of cardiovascular risk factors and inflammation, while corticosteroid use emerges as an additional adverse factor.

Other authors have shown that that arterial disease as stenosis an occlusion has its signature in the impedance signal. Indeed, the increase in stenosis severity causes a diminution of the magnitude of the impedance signal but no change on the signal morphology has been detected [58].

## Heart failure

Authors propose a simple model of exclusively noninvasive measures, combining leg bioimpedance with history of myocardial infarction, age, and sex provides accurate predictive capacity. Leg bioimpedance is inversely associated with heart failure incidence in the general population [59].

Lung impedance appears to be useful for guiding heart failure treatment in patients with ST-elevation myocardial infarction and improving outcomes in outpatients with heart failure. Furthermore, bioelectrical impedance has potential as a noninvasive, quantitative heart failure variable for population-based research [60].

The diagnosis of HF can be aided with use of analyzing the effect of the Valsalva maneuver on non-invasively measured CO among patients admitted to the emergency department with undifferentiated dyspnea. CO change with VM was the most accurate exam in identifying congestive heart failure as the cause of dyspnea [61].

Jurek et al. have shown that ICG is a useful method in evaluating individual hemodynamic profiles in patients with acute decompensated heart failure [62].

The evaluation of hemodynamic parameters, especially thoracic fluid content obtained by ICG, seems to be a worthwhile addition to standard diagnostics, both at the stage of hospital admission and while monitoring the effects of treatment. ICG is a useful method in evaluating individual hemodynamic profiles in patients with acute decompensated heart failure [63].

González-Islas et al. investigated body composition changes by bioelectrical impedance vectorial analysis in subjects with and without right heart failure and evaluated the risk factors for devolvement of cardiac cachexia in HF subjects. They showed that right heart failure gives greater disturbances in body composition and is a risk factor to development cardiac cachexia [64].

Edema index (extracellular water-to-total body water ratio) determined using BIA could be a useful marker for HF severity that could predict future HF-related admissions in adult patients with congenital heart disease [65].

Krezenski presents the possible application and importance of use of noninvasive bioimpedance methods for remote (home-based) monitoring of patients with HF [66].

Bernal-Ceballos studied the level of agreement between single and multiple frequency bioelectrical impedance analysis devices and demonstrated good agreement for measurement of the R parameter. Likewise, the agreement in all classifications by the BIVA method was almost perfect [67].

## Cushing’s disease

Assessment of early cardiovascular hemodynamic dysfunction with ICG can be performed for diverse diseases such as Cushing's Disease. Cushing's disease is a rare condition associated with a high cardiovascular risk and hypercortisolemia-related hemodynamic dysfunction. ICG parameters in the Cushing's disease group showed: lower values of stroke index, cardiac index, velocity index, Heather index, and thoracic fluid content and a higher systemic vascular resistance index than those in the control group [68].

## Limitations

The ICG measurements can be affected by the biological composition, respiration, noise due to movement or equipment, blood circulation, volume blood from the transthoracic region, electrodes configuration and types [6]. The papers and studies presented in the previous section each focus on a given CVD and not on all CVDs. Similarly, there is not much work on the automatic diagnosis of CDVs by ICG.

However, the works exploiting the ECG signal are very numerous. Shah et al. counted 2634 patents linking CVDs to the ECG signal [69]. Machine learning and data mining techniques [69, 70] and deep learning [71] are widely used for automatic diagnosis based on the ECG signal.

The main handicap from our point of view is the absence of a database of bioimpedance signals similar to that of ECG signals [72]. The only databases available are: Bp-graphene-bioimpedance [73], and for water composition to quantitative dehydration estimation [74].

There is an urgent need to build a database which, for each CDV, records the bioimpedance signal as well as the diagnosis obtained by a reference method. Such a database will first allow us to study the possibility of detecting each of the diseases by the bioimpedance method. It will also allow us to design a platform for the automatic detection of CDVs, in particular by exploiting the artificial intelligence techniques.

Although bioimpedance measurement technology is a mature method, a major research effort must be made in conjunction with experts in clinical diagnosis to improve the sensitivity, the specificity, and the confidence in the use of this technology in routine clinical practice. The data provided in most of the studies are small and often are not controlled. More extensive studies must be conducted to test the clinical utility of this technology and extend its application beyond the scope of the research [7].

## Conclusion

CVDs have a high cost in human and material terms. Prevention and early detection can help with better management. The bioimpedance method has proven to be effective and has allowed the non-invasive detection of several CDVs at a lower cost. In order to fully assess its diagnostic capabilities for various CDVs, it is necessary to build a database of ICG signals. This database will allow us to design an automatic detection system for CDVs.
